# Supplementary material for: Compound climate events transform electrical power shortfall risk in the Pacific Northwest
Source: Nat Commun. 2019 Jan 2;10:8. doi: 10.1038/s41467-018-07894-4 (PMC6315041; doi:10.1038/s41467-018-07894-4)
Supplement: Supplementary file 1 — Supplementary Information [file 41467_2018_7894_MOESM1_ESM.pdf]

Compound climate events transform electrical power shortfall risk in the Pacific Northwest

S.W.D. Turner, N. Voisin, J. Fazio, D. Hua, & M. Jourabchi

Supplementary Information

Supplementary Figures

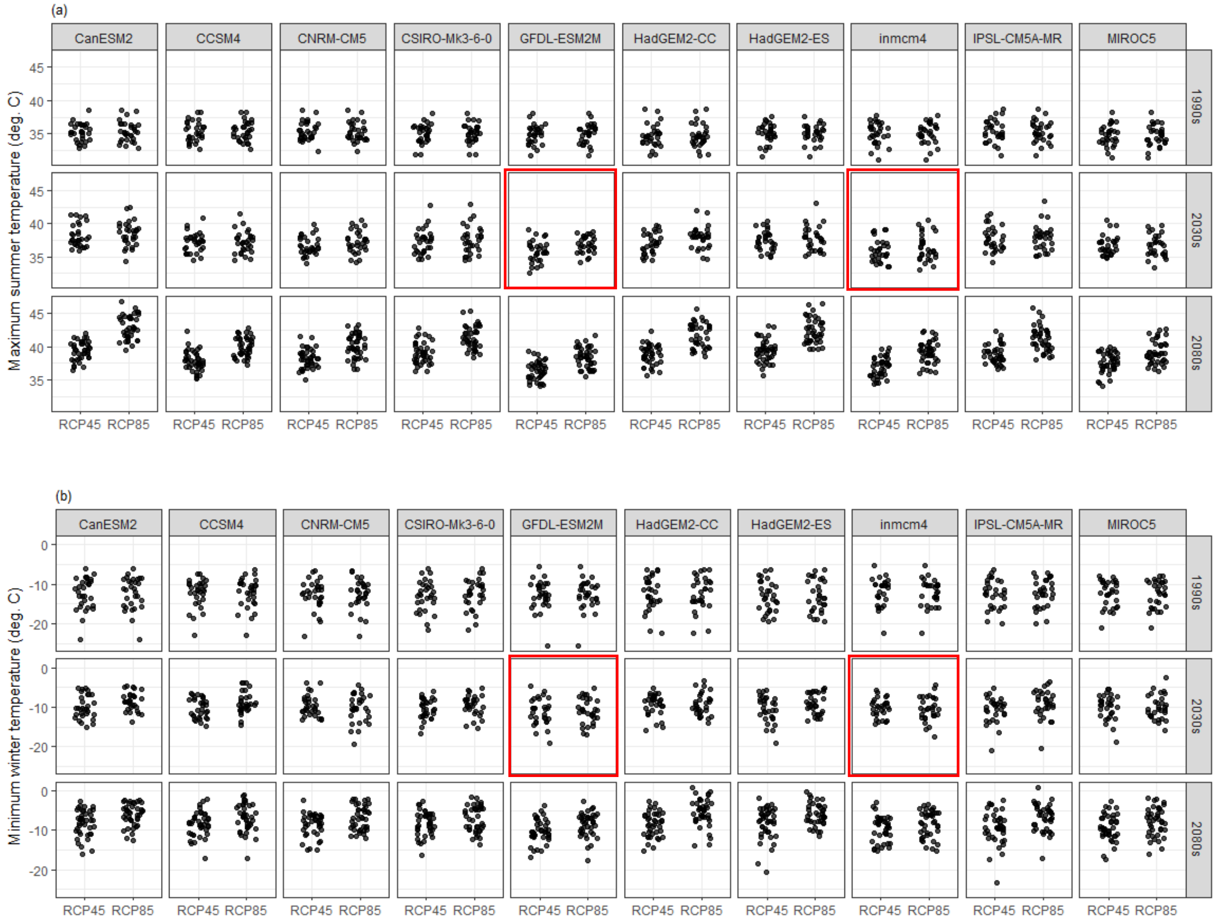

Supplementary Figure 1 – Impacts of climate change on temperature extremes. Data are from projected annual (summer) maximum and (winter) minimum temperatures for three time-slices, ten climate models, and two representative concentration pathways. Time slides are 30 years (1980 – 2009, 2020 – 2049, and 2060 – 2099), averaged spatially over the Pacific Northwest. Each point represents an annual maximum / minimum temperature. Maximums are computed from daily maximum temperatures whilst minimums are computed from daily minimum temperatures. Climate models selected in this study are highlighted.

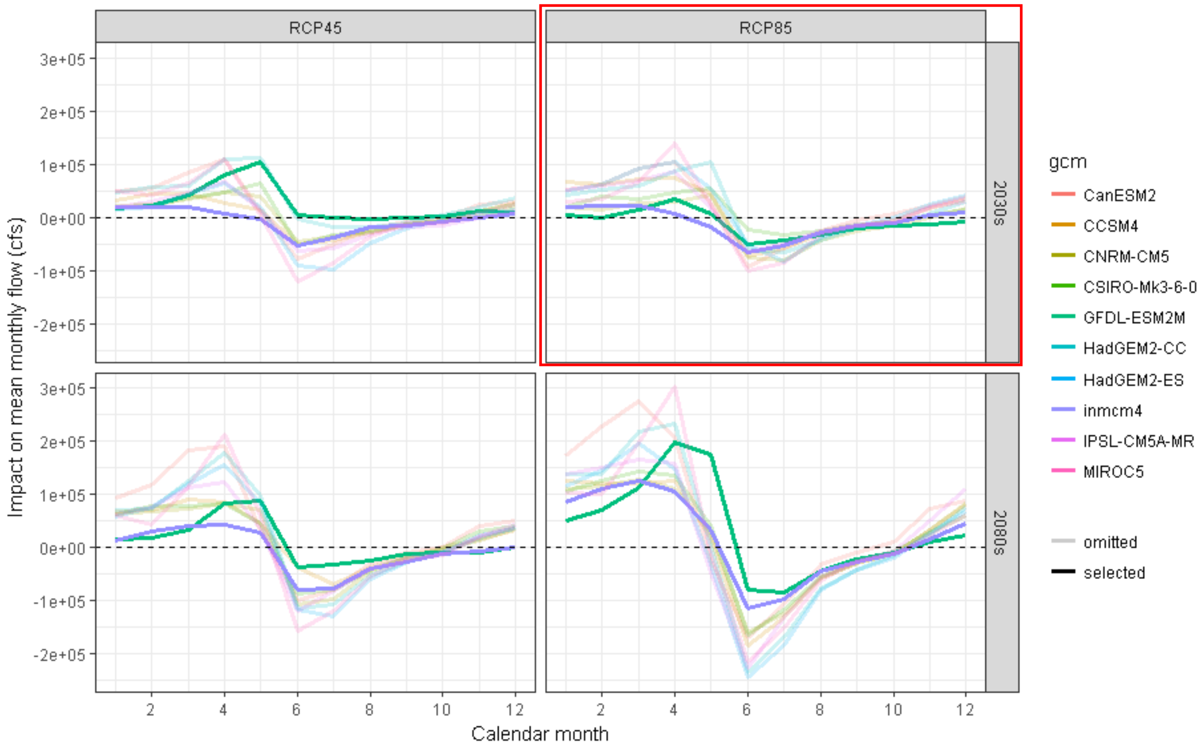

Supplementary Figure 2 – Impact of climate change on streamflow. Results based on simulated, unmodified monthly average streamflow at The Dalles, Columbia River. Impact on streamflow is computed from future average monthly streamflow for 2020 – 2049 relative to historical time slice of 1980 – 2009. Selected models (shown without color fade) are GFDL-ESM2M and INMCM4.

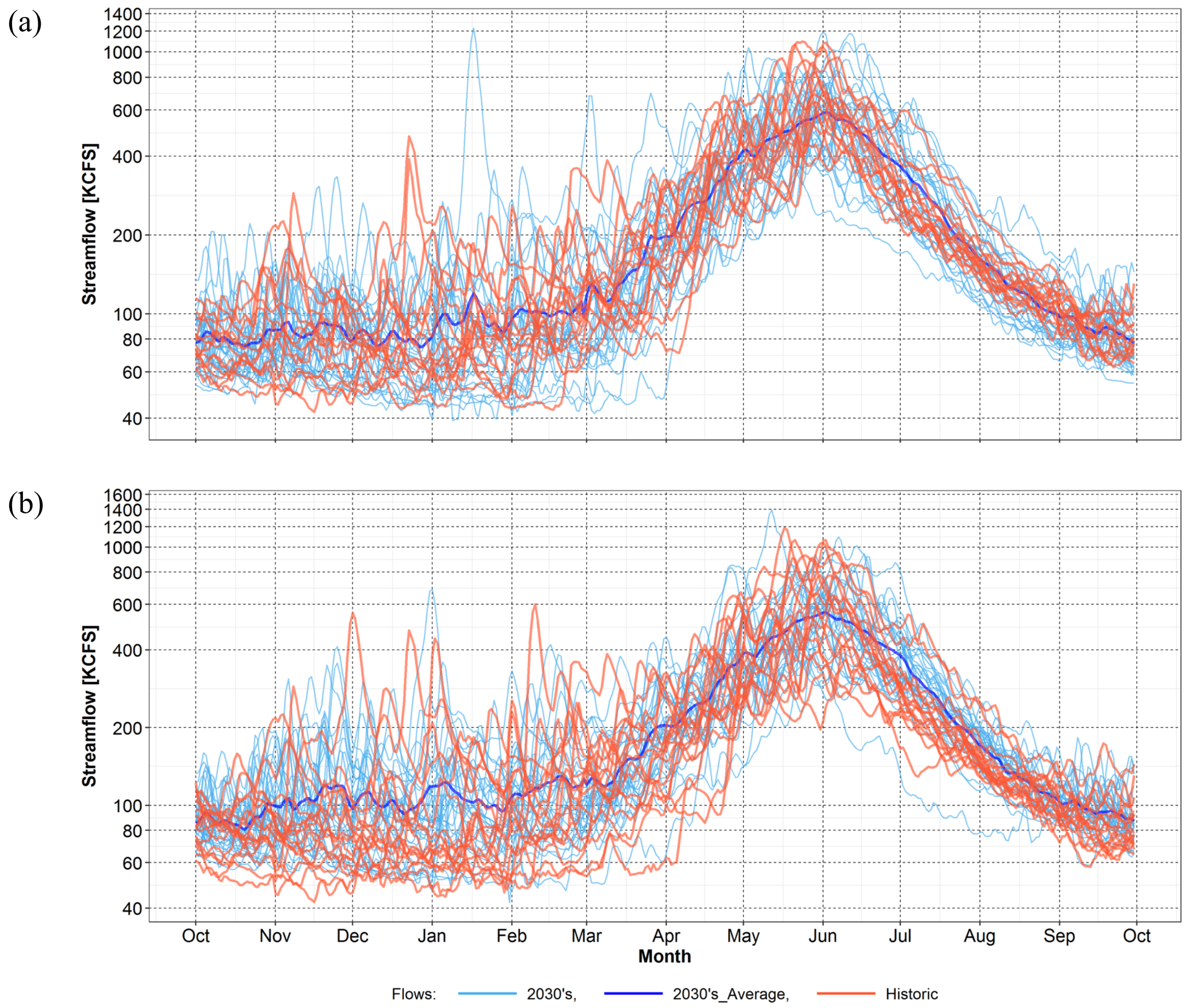

Supplementary Figure 3 – Selected water years for the two selected models. Results given for (a) GFDL-ESM2M and (b) INMCM4 climate forcing. The historical water years used to represent average future flow conditions (and associated occurrence weights—not shown) are selected by optimization, with the objective of selecting those years that minimize the root-mean-squared-error between historical and 2030s average monthly flows.

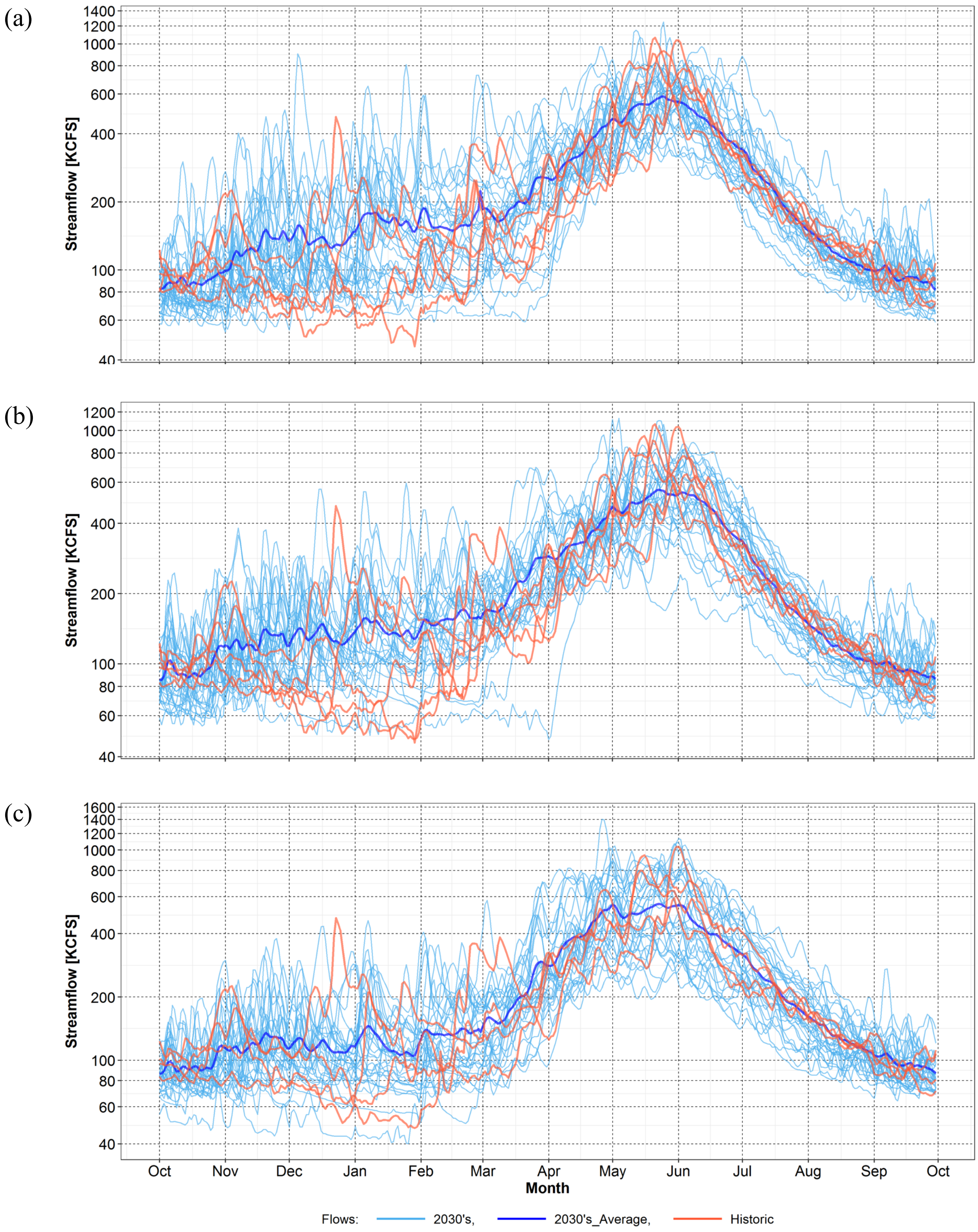

Supplementary Figure 4 - Selected water years for three omitted models. Results given for (a) CCSM, (b) HadGEM, (c) MIROC climate forcing. The historical water years used to represent average future flow conditions (and associated occurrence weights—not shown) are selected by optimization, with the objective of selecting those years that minimize the root-mean-squared-error between historical and 2030s average monthly flows.

Supplementary Tables

Supplementary Table 1 – Power system adequacy metrics. These are calculated for 2035 infrastructure for existing and carbon risk policy scenarios, and under no climate change and with climate change projected by two global atmospheric models (GFDL-ESM2M, INMCM4). Metrics are Loss of Load Probability (LOLP), Expected Unserved Load (EUE), Loss of Load Hours (LOLH), Average Maximum Shortfall (AMS), and Average Event Duration (AED).

(a) Existing resource expansion policy

		GFDL-ESM2M			INMCM4		
	Current climate	Loads only	Hydro only	Combined impacts	Loads only	Hydro only	Combined impacts
LOLP (%)	4.6	4.5	6.7	10.2	3.9	5.8	9.3
EUE (MWh)	1192	170	1497	370	136	1115	337
LOLH (Hr)	1.04	0.46	1.45	0.97	0.37	1.10	0.92
AMS (MW)	1009	393	886	409	400	829	391
AED (Hr)	13.8	6.3	12.8	6.4	6.3	11.6	6.3

(b) Carbon risk resource expansion policy

		GFDL-ESM2M			INMCM4		
	Current climate	Loads only	Hydro only	Combined impacts	Loads only	Hydro only	Combined impacts
LOLP (%)	3.8	1.6	4.8	4.0	1.4	4.2	4.0
EUE (MWh)	1007	84	1299	213	65	1098	389
LOLH (Hr)	0.93	0.19	1.30	0.47	0.15	1.16	0.62
AMS (MW)	952	415	935	410	424	816	628
AED (Hr)	12.6	7.0	13.6	7.5	7.4	11.8	8.37
